# Sphingolipid Signaling in Vascular Smooth Muscle Cells during Development and Diseases

**DOI:** 10.31662/jmaj.2025-0594

**Published:** 2026-03-19

**Authors:** Irusha Dahal, Christine Sanderson, Junichi Saito

**Affiliations:** 1 Vascular Biology Center, Medical College of Georgia at Augusta University, Augusta, GA, USA

**Keywords:** smooth muscle, sphingolipids, sphingosine-1-phosphate receptors, sphingosine kinase, vascular remodeling

## Abstract

Sphingolipids are essential components of the cell membrane that can be metabolized into bioactive metabolites such as sphingosine-1-phosphate (S1P), which function as signaling molecules both inside and outside cells. S1P primarily regulates cell survival, proliferation, migration, and adhesion by binding to S1P receptors (S1PRs). Sphingolipid signaling is essential for normal cardiovascular development, with impaired S1P production or defective S1PRs leading to embryonic lethality in mice due to vascular defects. Abnormal activation of sphingolipid signaling in pathologies such as atherosclerosis and pulmonary hypertension leads to excessive proliferation and migration of vascular smooth muscle cells (VSMCs). Pharmacological inhibition of the S1PR pathway is clinically available. By outlining current knowledge on sphingolipid signaling in VSMCs and its role in associated vascular diseases, this review intends to highlight the need to advance research targeting VSMCs as a potential therapeutic option for cardiovascular diseases.

## Introduction

Sphingolipids were initially identified as cell membrane components and recognized for their role in maintaining membrane structure. Subsequent studies have revealed that sphingolipids and their derivatives mediate complex signaling pathways with diverse biological activities ^(1)^. Among these sphingolipids, sphingosine-1-phosphate (S1P) serves as a key mediator in processes such as cell survival, proliferation, and migration ^(2)^. Sphingosine kinase (SPHK), an enzyme often dysregulated in disease states, synthesizes S1P and thereby plays a critical role in regulating cell fate. Following its synthesis, S1P activates a family of five high-affinity G-protein-coupled receptors, termed S1P receptors (S1PRs), to drive diverse cellular signaling ^(3)^.

During embryogenesis, the circulatory system is the first organ to develop; appropriate proliferation and migration of vascular smooth muscle cells (VSMCs) are essential for cardiovascular development ^(4)^. Furthermore, VSMC proliferation and migration are implicated in pathologies such as atherosclerosis, pulmonary hypertension, and restenosis following angioplasty ^(5)^. Unlike other muscle cells (e.g., skeletal and cardiac muscle cells), VSMCs do not undergo terminal differentiation and retain high cellular plasticity, allowing them to switch between contractile and synthetic phenotypes ^(6), (7)^. Contractile, differentiated VSMCs express contractile proteins and rarely proliferate, whereas synthetic, dedifferentiated VSMCs exhibit high proliferative capacity in response to environmental stimuli. For example, in response to vascular injury, VSMCs downregulate SMC-specific contractile genes and transition to a dedifferentiated, synthetic, and proliferative phenotype, leading to neointimal hypertrophy. The dynamic balance between these contractile and synthetic phenotypes plays a crucial role in both physiological vascular development and pathological vascular diseases ^(4), (5), (6), (7)^.

Mice with impaired S1P production (i.e., *Sphk1^(-/-)^*, *Sphk2^(-/-)^* mice ^(8)^) or defective S1PRs (i.e., *S1pr1^(-/-)^* mice ^(9)^ and *S1pr1^(-/-)^, S1pr2^(-/-)^, S1pr3^(-/-)^* mice ^(10)^) exhibit embryonic lethality due to vascular defects, such as impaired VSMC recruitment, indicating that sphingolipid signaling is essential for normal cardiovascular development. Additionally, sphingolipid signaling regulates VSMC proliferation and migration in pathologies such as pulmonary hypertension ^(11), (12)^. However, despite advances in research on sphingolipid signaling in endothelial cells (ECs) and immune cells, many aspects of sphingolipid signaling in VSMCs remain unclear. Therapeutic approaches targeting sphingolipid signaling have already been adopted in clinical practice for immune diseases ^(13)^, but have not yet used in cardiovascular diseases. Therefore, further elucidation of sphingolipid signaling in VSMCs has implications for the development of novel therapeutic approaches for cardiovascular diseases. Accordingly, this review focuses on sphingolipid signaling in VSMCs and vascular diseases, with particular emphasis on VSMC biology.

## SPHKs

**Figure 1.**
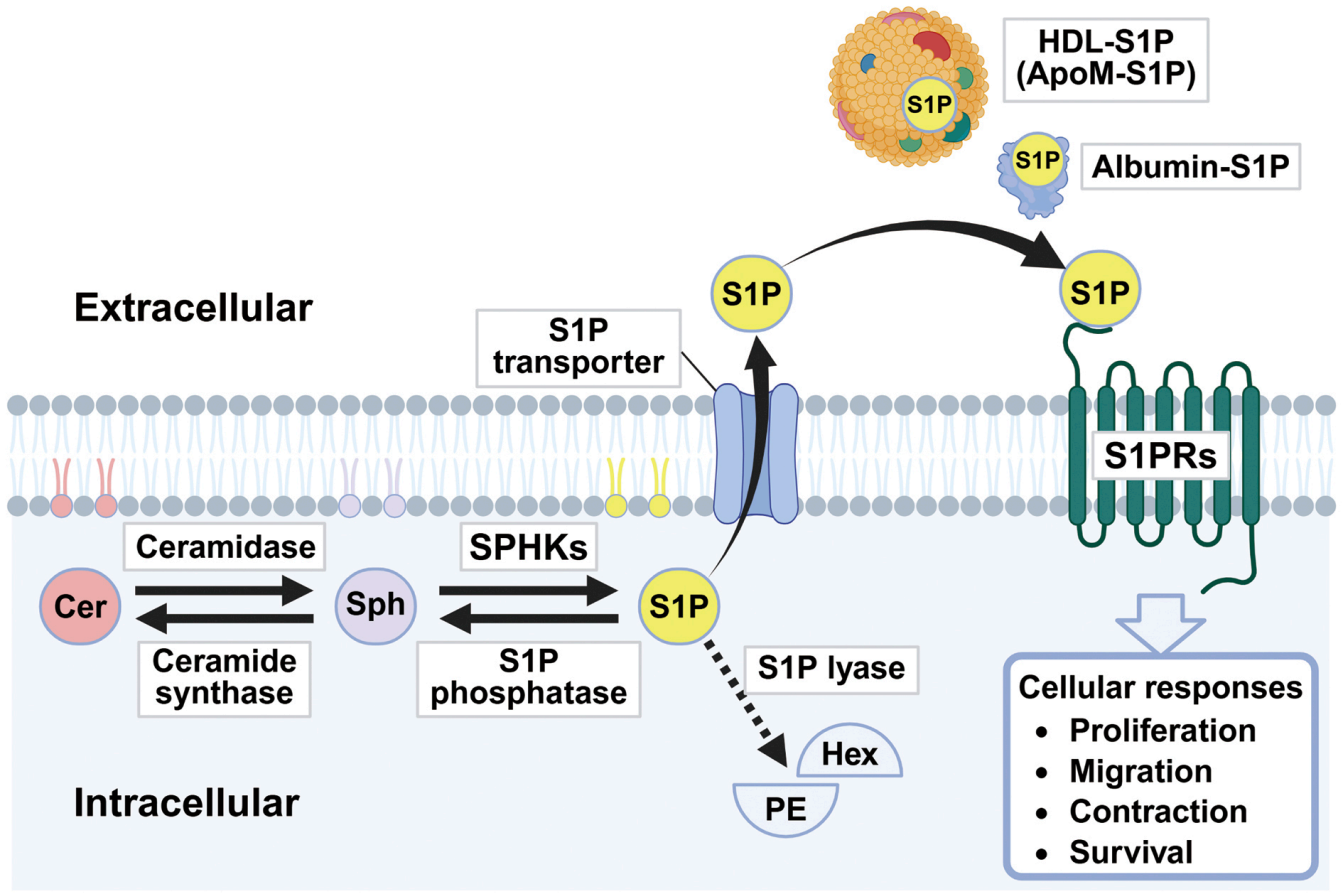
Biosynthesis and transport of S1P. SPHKs phospholylate sphingosine (Sph) to S1P. S1P is secreted into the extracellular space via the S1P transporter. Chaperone proteins (i.e., Apolipoprotein M [ApoM] and Albumin) enable S1P transport. Extracellular S1P binds to S1PRs on cell membranes via autocrine or paracrine mechanisms. S1P binding to S1PRs initiates cellular responses via G-proteins, influencing cell proliferation, migration, contraction, and survival. Graphical objects were created with BioRender (https://biorender.com/). Cer: ceramide; HDL: high-density lipoprotein; Hex: hexadecenal; PE: phosphoethanolamine; SIP: sphingosine-1-phosphate; SIPR: sphingosine-1-phosphate receptor; SPHK: Sphingosine kinase.

The biologically active S1P is a key mediator in processes such as cell survival, proliferation, and angiogenesis ^(2)^ (Figure 1). SPHK, which catalyzes the phosphorylation of sphingosine to generate S1P, has two isoforms, SPHK1 and SPHK2, derived from distinct genes located on chromosomes 17q25.2 and 19q13.2, respectively ^(2)^. At the structural level, SPHK1 and SPHK2 exhibit high homology; however, SPHK2 possesses a nuclear localization sequence at its N-terminus and a nuclear export sequence at its C-terminus. Thus, SPHK2 is capable of translocating between the cytoplasm and nucleus, whereas SPHK1 activity is confined to the cytoplasm ^(14)^. In response to stimuli such as growth factors, SPHK1 can be recruited to the cell membrane to generate S1P ^(2)^. The generated S1P is then released into the extracellular space and binds to S1PRs to modulate downstream signaling ^(2)^ (Figure 1). Conversely, SPHK2 is primarily localized to the endoplasmic reticulum and can enter and exit the nucleus ^(15)^. SPHK2 activity within the nucleus inhibits both DNA synthesis, causing cell cycle arrest ^(16)^, and histone deacetylases, altering the epigenetic regulation of gene expression ^(17)^. Knockout mouse models for either SphK1 or SphK2 develop normally; however, genetic deficiency of both isoforms causes fetal death due to abnormal angiogenesis and severe hemorrhage ^(8)^. Therefore, SphK1 and SphK2 are considered to possess complementary, redundant functions.

Increased expression and activation of SPHK1 in VSMCs have been reported in both human patients with pulmonary arterial hypertension and mouse models of the disease (discussed later) ^(11), (12)^. Furthermore, defective elastic fibers increase SPHK1 levels, leading to excessive VSMC proliferation ^(18)^. Pharmacological or genetic inhibition of SPHK1 can improve the phenotype in mouse models of these diseases, suggesting that inhibition of abnormally increased SPHK1 is a potential therapeutic strategy for vascular diseases characterized by VSMC accumulation.

## S1P

Sphingosine-1-phosphate levels are regulated through four stages: production, degradation, transport into the extracellular space, and movement within the extracellular space (Figure 1).

SPHK1 and SPHK2 are responsible for S1P production. S1P is undetectable in *Sphk1^(-/-)^*, *Sphk2^(-/-)^* mice, suggesting that S1P is produced exclusively by SPHKs ^(8)^. S1P degradation is regulated through its irreversible breakdown into hexadecenal and phosphoethanolamine by S1P lyase, as well as through its reversible dephosphorylation by S1P phosphatases or a family of broad-specificity lipid phosphate phosphatases (Figure 1) ^(19)^. S1P levels are generally low in tissues because it is rapidly degraded by S1P lyase, whereas plasma S1P levels are relatively high ^(20)^.

In ECs, S1P is exported from the cytoplasm to extracellular compartments via Spinster Homolog 2 (Spns2), as demonstrated in experiments with EC-specific *Spns2-*knockout mice (*Spns2^(flox/flox)^*, *Tie2-Cre*) ^(21)^. In erythrocytes and platelets, S1P export occurs via major facilitator superfamily transporter 2b ^(22)^. However, the specific transporter involved in VSMCs has not yet been fully evaluated.

Because of the limited water solubility of S1P, chaperone proteins bind to S1P, enabling its transport in both circulating and interstitial fluids (Figure 1). A representative S1P chaperone is apolipoprotein M, a component of high-density lipoprotein (HDL) ^(23)^. Approximately 60-80% of S1P in human plasma is bound to HDL. Serum albumin also binds to S1P with low affinity and functions as a chaperone protein. Changes in serum S1P levels may indicate disease severity in individuals with obstructive coronary artery disease and atherosclerosis ^(24), (25), (26)^. This extracellular S1P binds to S1PR, activating various cellular signaling pathways (described below).

## S1PRs

The majority of S1P effects are mediated through activation of S1PRs on cell membranes via autocrine or paracrine mechanisms ^(27), (28)^ (Figure 1). S1PRs (S1PR1-S1PR5) represent a G-protein-coupled receptor family. In physiological adult blood vessels, S1PR1 is primarily expressed in vascular ECs, whereas S1PR2 and S1PR3 are mainly expressed in VSMCs ^(29)^. S1PR4 is primarily expressed within immune compartments and leukocytes, whereas S1PR5 is mainly expressed in the central nervous system ^(27)^. S1P exerts diverse effects across organs depending on the relative expression of receptor subtypes and the availability of S1P to receptors ^(30)^.

**Figure 2.**
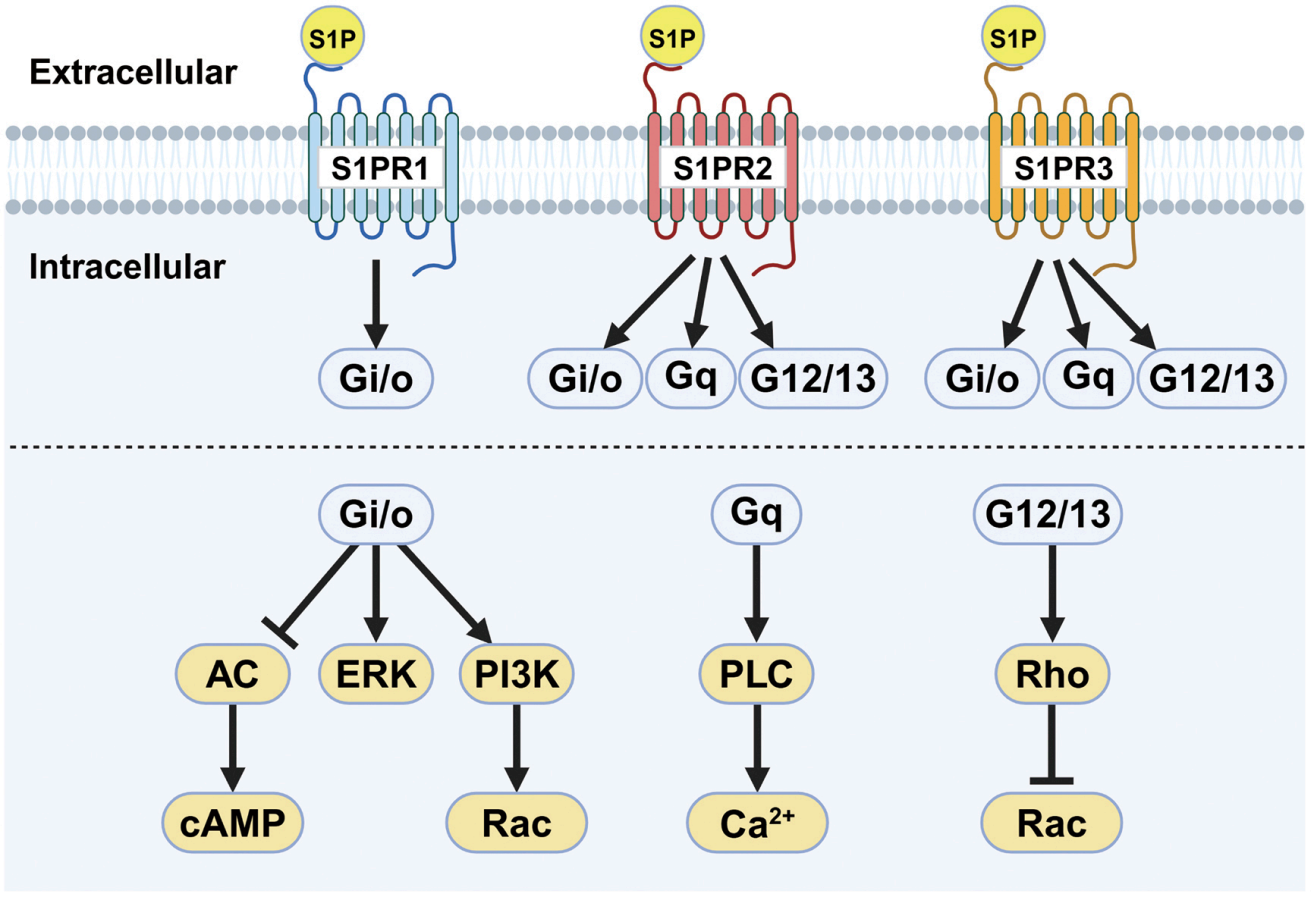
S1P receptors (S1PRs) and downstream signaling. S1PRs are G protein-coupled receptors. The binding of S1P to S1PRs leads to the activation of G-proteins (Gi/o, Gq, G12/13). G-proteins regulate the activation or inhibition of the downstream signaling pathways. Graphical objects were created with BioRender (https://biorender.com/). AC: adenylyl cyclase; Camp: cyclic adenosine monophosphate; ERK: extracellular signal-regulated kinases; PI3K: phosphatidylinositol-3 kinase; PLC: phospholipase C; SIP: sphingosine-1-phosphate.

Different S1P signaling pathways mediated by distinct S1PRs result from their diverse and sometimes overlapping coupling to G proteins (Figure 2). S1PR1 binds exclusively to Gi/o, whereas S1PR2 and S1PR3 bind to Gi/o, Gq, and G12/13 ^(31)^. The specific G protein to which S1PR binds leads to distinct intracellular signaling pathways and cellular responses (Figure 2). Gi/o primarily affects adenylate cyclase, with Gi/o activation causing a decrease in cyclic adenosine monophosphate ^(32)^. Gi/o also induces activation of protein kinase C isoforms α and ε, extracellular signal-regulated kinases 1/2 signaling, and the phosphatidylinositol-3 kinase/protein kinase B pathway ^(33), (34)^. Gq activation induces phospholipase C, which increases intracellular Ca^2+^, whereas G12/13 inhibits downstream Rac via Rho activation ^(35), (36)^.

A recent study demonstrated that β-catenin regulates S1PR1 expression in VSMCs ^(37)^. SMC-specific β-catenin-knockout mice (i.e., *Ctnnb1^(flox/flox)^*, *Acta2-CreER^T2^* mice) showed reduced S1PR1 expression in aortic SMCs and attenuated intimal hyperplasia in the carotid artery after ligation ^(37)^. *S1pr1* overexpression under the *Acta2* promoter increased carotid ligation-induced intimal thickening in SMC-specific β-catenin-knockout mice ^(37)^. Furthermore, this study performed a partial ligation of the carotid artery in apolipoprotein E mutant mice (*ApoE^(-/-)^* mice), which exhibit high plasma cholesterol levels on a high-fat diet, to generate turbulence-induced atherosclerosis. Administration of E7386, an inhibitor of the C-terminal region of β-catenin, then reduced atherosclerosis ^(37)^. These results suggest the potential of targeting mediators of S1PR expression in a cell-type-specific manner as a therapeutic approach.

### S1PR1

During embryogenesis in murine models, VSMCs are first observed on the ventral side of the aorta at embryonic day (E) 10.5, and then migrate to the dorsal side ^(38)^. VSMCs completely cover the aorta by E11.5. *S1pr1^(-/-)^* embryos exhibit deficient VSMCs coverage at E12.5, but normal EC morphology at E11.5 ^(9)^. These *S1pr1^(-/-)^* embryos exhibited lethality caused by severe bleeding between E12.5 and E14.5 ^(9)^. Another study found that VSMCs from rat neonates exhibit greater proliferative capacity and higher S1PR1 expression than those from adult rats ^(39)^. After transduction of S1PR1 using adenoviral infection, rat adult VSMCs showed increased migration in response to exogenous S1P ^(39)^.

In adults, VSMCs generally express low levels of S1PR1. However, certain stimuli, such as vascular injury, can induce S1PR1 expression in VSMCs. In the intact carotid artery of adult mice, S1PR1 expression was restricted to ECs; however, after ligation, S1PR1 was detected in both ECs and the region of intimal thickening ^(40)^. Moreover, mice with *S1pr1* overexpression under the *Acta2* promoter exhibited severe hyperplasia compared to wild-type mice ^(40)^. VSMCs isolated from these mice showed greater proliferative capacity than those from wild-type mice ^(40)^. Another study demonstrated that the mRNA levels of S1PR1 and S1PR3 increased 48-72 hours after balloon injury in the carotid artery of rats, whereas S1PR2 mRNA expression decreased ^(41)^. Furthermore, administration of VPC44116, an inhibitor of S1PR1 and S1PR3, improved intimal hyperplasia following balloon injury ^(41)^. *In vitro* experiments showed that VPC44116 inhibited VSMC proliferation, whereas JTE013, an S1PR2 inhibitor, promoted VSMC proliferation ^(41)^. Another study showed that S1P stimulation of rat neonatal VSMCs activates S1PR1, leading to increased expression of platelet-derived growth factor-A and -B chains, which are chemotactic attractants ^(42)^.

### S1PR2

Sphingosine-1-phosphate receptor 2 is the primary receptor expressed in adult VSMCs. In contrast with S1PR1, S1PR2 activation can inhibit VSMC migration. In *S1pr2*-knockout mice (*S1pr2^(-/-)^* mice), intimal thickening after carotid artery ligation was enhanced compared to that in wild-type mice ^(43)^. Furthermore, VSMCs isolated from the carotid arteries of *S1pr2*-knockout mice exhibited increased cell migration following treatment with S1P alone or with the combination of S1P and PDGF, compared to VSMCs from wild-type mice in vitro ^(43)^. S1PR2 activates Rho by coupling to G12/13, which inhibits cell migration ^(36)^. Consistent with this, exogenous S1P activates Rho in wild-type VSMCs but not in *S1pr2^(-/-)^* VSMCs ^(43)^. Another study found that, following carotid wire injury, FVB mice formed significant intimal thickening, whereas C57BL/6 mice developed only minor lesions ^(44)^. The authors demonstrated that VSMCs isolated from the carotid arteries of FVB mice showed high S1PR1 expression and increased cell migration in response to S1P administration, whereas VSMCs isolated from the carotid arteries of C57BL/6 mice showed high S1PR2 expression and decreased cell migration in response to S1P administration ^(44)^. Experiments using human aortic SMCs and rat aortic SMCs also demonstrated that S1PR2 activation inhibits VSMC migration by suppressing Rac activity ^(45)^. Moreover, treatment of VSMCs with S1P increases the expression of SMC differentiation markers, including SMα-actin, SM myosin heavy chain, and SM22 ^(46)^. Experiments using selective S1PR antagonists demonstrated that signaling via S1PR2 upregulates SMC differentiation markers, whereas signaling via S1PR1 and S1PR3 downregulates these genes ^(41)^.

### S1PR3

Sphingosine-1-phosphate receptor 3 is more highly expressed in the iliac-femoral arteries than in the carotid arteries ^(47)^. Shimizu et al. ^(47)^ performed iliac-femoral artery denudation in *S1pr3^(-/-)^* mice or wild-type mice and found that *S1pr3^(-/-)^* mice exhibited less SMC proliferation and attenuated intimal thickening compared to wild-type mice 28 days after surgery. *S1pr3* overexpression in VSMCs isolated from the carotid artery of *S1pr3^(-/-)^* mice leads to increased cell proliferation and migration in vitro ^(47)^. Conversely, Keul et al. ^(48)^ performed carotid ligation in *S1pr3^(-/-)^* and wild-type mice and found that *S1pr3^(-/-)^* mice exhibited greater intimal thickening than wild-type mice 28 days after surgery. These conflicting results may be explained by differences in S1PR3 expression levels across different vascular beds or by the presence or absence of residual ECs with different surgical procedures.

## Role of Sphingolipid Signaling in VSMCs in Arterial Diseases

**Figure 3.**
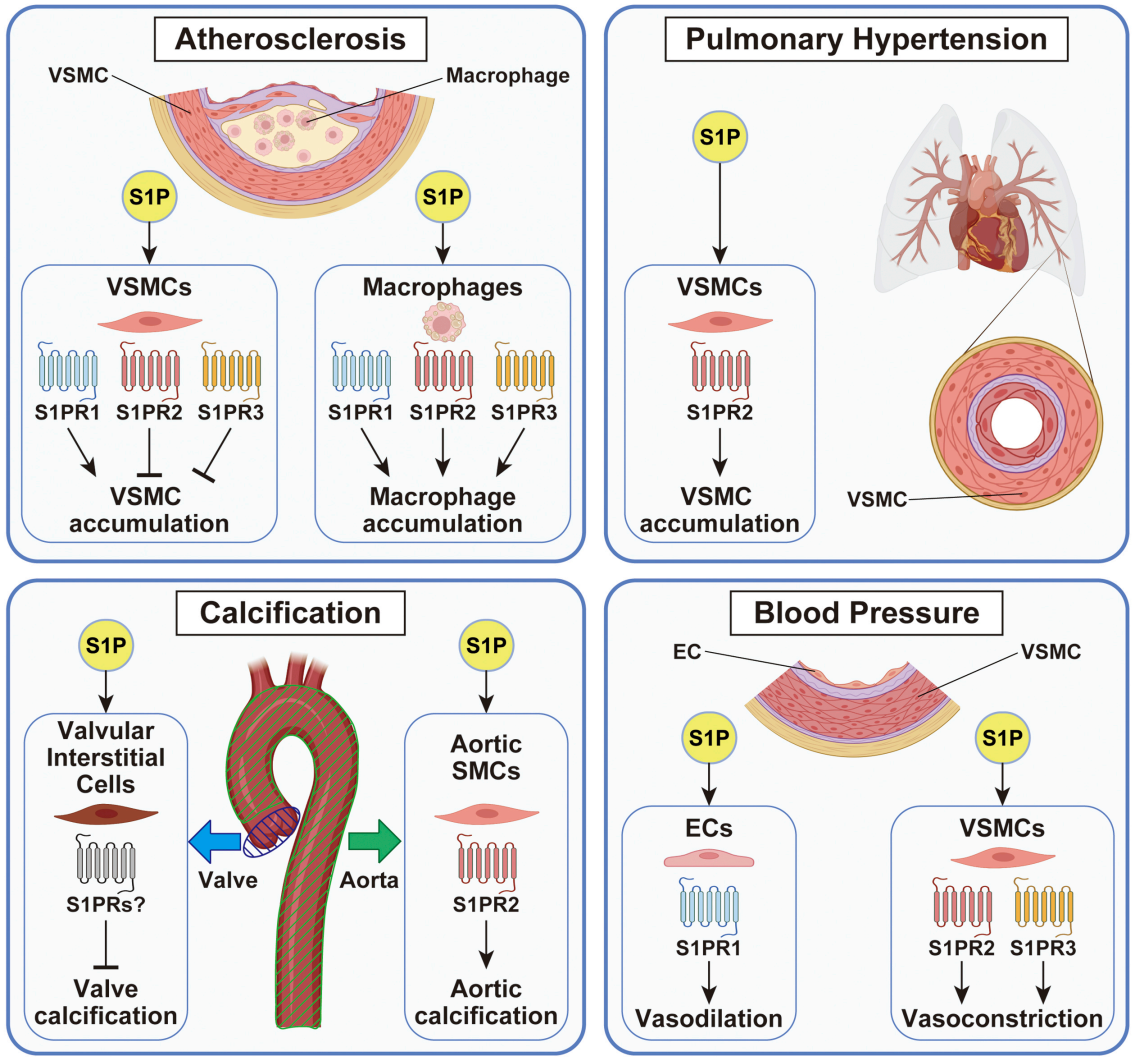
Sphingolipid signaling regulates vascular disease and homeostasis. Examples of the influences of S1P-S1PRs signaling on vascular diseases and homeostasis. Even the same receptors can have different effects depending on the type of disease, vascular bed, or cell type. Graphical objects were created with BioRender (https://biorender.com/). EC: endothelial cell; S1P: sphingosine-1-phosphate; S1PR: S1P receptor; VSMC: vascular smooth muscle cell.

Here, we review studies focusing on VSMCs in atherosclerosis, pulmonary hypertension, calcification, and blood pressure homeostasis (Figure 3).

### Atherosclerosis

Atherosclerosis is a chronic systemic disease affecting various vascular beds, characterized by plaque formation in the vessel wall, macrophage infiltration, and luminal narrowing caused by VSMC accumulation. Although multiple cell types intricately contribute to the development of atherosclerosis, the precise role of sphingolipid signaling in this disease remains incompletely understood. However, as shown below, multiple studies have suggested sphingolipid signaling as a novel therapeutic target.

Low HDL levels are associated with a higher risk of atherosclerosis and heart disease. Because HDL serves as a chaperone for S1P in blood, studies have sought to elucidate the relationship between serum S1P levels and coronary artery disease. Deutschman et al. ^(24)^ analyzed serum S1P levels in patients undergoing cardiac catheterizations for all indications (n = 308) and revealed that higher S1P levels are associated with the prevalence of obstructive coronary artery disease (CAD). In contrast, Soltau et al. ^(26)^ reported that serum S1P levels in patients with atherosclerosis (n = 132) were lower than those of healthy blood donors (n = 174). Sattler et al. ^(25)^ analyzed S1P levels in plasma and HDL from patients with stable CAD (n = 95), patients with myocardial infarction (n = 83), and healthy blood donors (n = 85) and found that total plasma S1P levels were lower in individuals with stable CAD than in controls. However, normalization to HDL revealed higher normalized S1P levels in patients with stable CAD and myocardial infarction than in healthy controls ^(25)^. The amount of non-HDL-bound S1P was also higher in individuals with stable CAD and myocardial infarction than in controls ^(25)^. These studies using clinical samples indicate that S1P distribution between HDL and non-HDL pools may be necessary for CAD.

Low-density lipoprotein receptor mutant mice (*Ldlr^(-/-)^* mice) have high blood cholesterol levels and exhibit atherosclerosis, especially when fed a western diet. *Ldlr^(-/-)^* mice that received bone marrow transplantation from S1P lyase mutant mice (*Sgpl1^(-/-)^* mice) and were fed a western diet containing 0.25% cholesterol exhibited high plasma S1P levels and reduced atherosclerotic lesions ^(49)^. Moreover, SKI-II, a SPHK1 inhibitor, worsened atherosclerotic lesions in mice fed a western diet containing 1.25% cholesterol ^(50)^. These studies indicate that increased S1P is protective against atherosclerosis.

In cell culture experiments, the inhibitory effect of HDL-bound S1P on VSMC migration was abolished by S1PR2-specific small interfering RNA, but enhanced in S1PR2-overexpressing cells, suggesting that S1PR2 is involved in suppressing VSMC migration by HDL-bound S1P ^(51), (52)^. However, a study using *ApoE^(-/-)^*, *S1pr2^(-/-)^* mice showed reduced atherosclerotic lesions ^(53)^. *ApoE^(-/-)^* mice that received bone marrow transplantation from *ApoE^(-/-)^*, *S1pr2^(-/-)^* mice also showed reduced atherosclerotic lesions ^(53)^. Moreover, attenuated inflammatory responses may explain these atheroprotective features. Lower macrophage accumulation was observed in atherosclerotic lesions in *ApoE^(-/-)^*, *S1pr2^(-/-)^* mice and *ApoE^(-/-)^* mice receiving *ApoE^(-/-)^*, *S1pr2^(-/-)^* bone marrow ^(53)^. *S1pr2^(-/-)^* mice also showed reduced levels of pro-atherosclerotic cytokines, such as interleukin (IL)-1β and IL-18, after lipopolysaccharide treatment ^(53)^. These studies indicate that activation of S1P-S1PR2 signaling may exert both pro- and anti-atherosclerotic effects by suppressing VSMC proliferation and promoting macrophage accumulation and inflammatory cytokine production.

As mentioned above, S1PR1 promotes the proliferation and migration of VSMCs. Furthermore, S1PR1 mediates macrophage migration ^(54)^. FTY720, a functional antagonist of S1PR1, attenuated atherosclerosis lesions in *ApoE^(-/-)^* mice fed a western diet containing 0.15% cholesterol ^(55)^. Similarly, atherosclerotic lesions were suppressed in *Ldlr^(-/-)^* mice fed with a Western diet containing 1.25% cholesterol ^(56)^. KRP-203, a selective S1PR1 antagonist, also attenuated atherosclerosis in *Ldlr^(-/-)^* mice fed with a Western diet ^(57)^.

In another study, *ApoE^(-/-)^*, *S1pr3^(-/-)^* mice fed a standard chow diet exhibited no differences in atherosclerotic lesion size but had reduced monocyte and macrophage contents in the lesion ^(48)^. As mentioned above, *S1pr3^(-/-)^* mice that underwent carotid ligation exhibited greater intimal thickening than control mice ^(48)^. These results suggest that S1PR3 simultaneously exerts a pro-atherosclerotic effect in macrophages and an anti-atherosclerotic effect in VSMCs, which may offset each other during atherosclerosis development.

### Pulmonary hypertension

Pulmonary arterial hypertension (PAH) is a severe and progressive disease characterized by elevated pulmonary artery pressure associated with increased vascular resistance. Chronic elevation of pulmonary artery pressure increases the load on the right ventricle, ultimately leading to right heart failure and death. Although functional vasoconstriction may be involved in the initial stage of the disease, the central pathophysiology in the later stage is vascular remodeling, characterized by thickening of the pulmonary artery wall. This is primarily due to the transition of VSMCs from a contractile to a proliferative phenotype, accompanied by VSMC proliferation and migration.

Several studies have demonstrated that sphingolipid signaling plays a crucial role in the pathogenesis of PAH. SPHK1 expression was increased in lung tissues and pulmonary arterial SMCs from patients with PAH ^(11)^. Similarly, S1P levels were upregulated in the lungs of patients with PAH, and levels of SPHK1 and S1P were elevated in the lungs of hypoxia-induced PAH models in mice and rats ^(11)^. *Sphk1^(-/-)^* mice showed attenuation of hypoxia-induced PAH, whereas S1P lyase mutant mice (i.e., *Sgpl1^(+/-)^*) showed worsening of PAH after six weeks of hypoxia exposure ^(11)^. Hypoxia-induced PAH was attenuated by SKI2, an inhibitor of SPHK1 and SPHK2, in rats, and by JTE013, an S1PR2 inhibitor, in mice ^(11)^. Another study reported that SLP7111228, an inhibitor of SPHK1, attenuated serum S1P levels and reduced PAH in rats exposed to Sugen5416 and hypoxia ^(12)^. Furthermore, PF-543, a selective inhibitor of SPHK1, reduced right ventricular hypertrophy in hypoxia-induced PAH mice ^(58)^.

A recent study demonstrated that SMC-specific *Sphk1* deletion (*Sphk1^(flox/flox)^*, *Tangln-Cre*) rescued PAH in mice exposed to hypoxia alone or to hypoxia and Sugen5416 ^(59)^. Furthermore, studies indicate that S1P binds to S1PR2 and promotes nuclear translocation of YAP in pulmonary artery SMCs, activating Notch3 and thereby promoting proliferation ^(59), (60), (61)^. These results indicate that a series of signals, including SPHK1, S1P, S1PR2, YAP, and Notch3, may represent potential therapeutic targets for PAH.

### Calcification

Sphingolipid signaling is associated with osteogenic differentiation and calcification. In cell culture media that induce calcification, SPHK1 expression is increased in VSMCs. Razazian et al. ^(62)^ recently reported that administration of SK1-IN-1, a SPHK1 inhibitor, or PF-543, a selective SPHK1 inhibitor, enhances osteogenic marker expression in aortic SMCs. When cholecalciferol was administered to induce medial vascular calcification, aortic calcification was more severe in *Sphk1^(-/-)^* mice than in wild-type mice ^(62)^. In contrast, SPHK1 inhibition improved aortic valve calcification in individuals with aortic valve stenosis ^(63)^. Benkhoff et al. ^(63)^ induced aortic valve stenosis via wire injury and demonstrated that calcification and valve stenosis were improved in *Sphk1^(-/-)^* mice with reduced S1P levels, whereas increased S1P levels induced by an S1P lyase inhibitor worsened aortic phenotypes. These effects were mediated by S1PR2 in valvular interstitial cells ^(63)^. Although valvular interstitial cells may have different characteristics from VSMCs, the results from these studies reflect the complexity of sphingolipid signaling in aortic and aortic valve calcification.

### Blood pressure homeostasis

The mechanisms of sphingolipid signaling in blood pressure homeostasis and in the pathophysiology of hypertension and hypotension are not fully understood. However, multiple studies suggest that S1P and S1PR are involved in blood pressure regulation ^(64)^.

S1P stimulates S1PR1 on ECs, activating endothelial nitric oxide synthase ^(65)^. The resulting nitric oxide relaxes VSMCs, reducing vascular tone. In fact, mice with EC-specific *S1pr1* deficiency (i.e., *Cdh5-CreER^T2^*, *S1pr1^(flox/flox)^* mice) show reduced EC-derived nitric oxide production and thus enhanced vasoconstriction, resulting in higher-than-normal blood pressure ^(66)^. These results suggest that S1PR1 activation in ECs contributes to VSMC relaxation, likely by activating endothelial nitric oxide synthase. Chronic administration of fingolimod, a functional S1PR1 inhibitor, reduces S1PR1 in ECs and increases systemic pressure ^(66)^. Conversely, exogenous S1P induces vasoconstriction by acting directly on VSMCs ^(67)^. In this regard, Salomone et al. ^(68)^ reported that the cerebral arteries of wild-type mice contract in response to S1P, whereas the basilar artery of *S1pr3^(-/-)^* mice shows a diminished response. Similarly, Szczepaniak et al. ^(69)^ reported that while pulmonary arteries of wild-type mice contract in response to S1P, pulmonary artery contraction is diminished in *S1pr2^(-/-)^* mice or wild-type mice treated with an S1PR2 antagonist. These results suggest that S1P may induce VSMC vasoconstriction by acting on S1PR2 and S1PR3 ^(64)^.

Most previous studies have performed pharmacological or genetic experiments on the entire vascular wall, including both ECs and VSMCs. Bioactive substances, including S1P, regulate vascular tone either by directly modulating VSMCs or by stimulating ECs to release nitric oxide, which then diffuses to VSMCs. Therefore, future studies using VSMC-specific genetically modified animals are crucial for elucidating these mechanisms in VSMCs.

## Potential Therapeutics

As shown above, sphingolipid signaling is involved in the pathogenesis of various vascular disorders. Based on these studies, the enzymes involved in S1P biosynthesis and S1PRs are considered promising therapeutic targets ^(70)^.

SPHKs are responsible for synthesizing S1P; however, no Food and Drug Administration (FDA)-approved therapeutic drugs currently target SPHKs. Possible reasons include low bioavailability, a short drug half-life, poor stability in vivo, and off-target effects ^(71)^.

In contrast to SPHKs, some S1PR-targeted therapies are available. Fingolimod, a functional antagonist of S1PR1, is the first S1PR-targeted drug approved by the FDA and has been used to treat patients with relapsing-remitting multiple sclerosis ^(13), (70)^. However, the use of fingolimod is restricted due to serious adverse events, including an initial decrease in heart rate and macular edema, caused by on-target effects of S1PR1 in the cardiac and retinal vasculature. Additionally, siponimod, which is more selective for S1PR1, has been approved by the FDA as a treatment for patients with relapsing-remitting multiple sclerosis; however, adverse events similar to those observed with fingolimod have also been reported.

Given these limitations, S1P chaperones also provide an alternative strategy for modulating S1P signaling. For example, therapeutic administration of artificially engineered apolipoprotein M-S1P reduced blood pressure in hypertensive mice, mitigated myocardial injury after ischemia/reperfusion injury, and decreased infarct volume in a cerebral infarction model ^(72)^. However, this approach is not yet clinically available.

## Current Limitations to Clinical Implementation for Vascular Diseases

There are currently limitations to the clinical translation of these therapeutic approaches for vascular diseases. The first challenge is the delivery of drugs to specific vascular beds and their VSMCs. Because most vascular diseases occur in specific vascular beds, such as the coronary and pulmonary arteries, effective strategies are needed to selectively target affected vasculatures while minimizing effects on other organs. In addition, the potential effects on ECs must be considered, as they form the innermost layer of blood vessels and are directly exposed to circulating therapies.

The second challenge is that targeting SPHKs and S1P chaperones induces widespread changes in S1P levels and availability, increasing the risk of off-target effects. In this context, given that specific S1PRs are involved in the pathogenesis of vascular diseases, selective inhibitors targeting a single S1PR represent a promising therapeutic strategy.

The third challenge is that the same enzymes, molecules, or receptors can exert distinct effects depending on the type of disease, vascular bed, or cell type. For example, S1PR2 in VSMCs may be protective against atherosclerosis ^(52)^, whereas it promotes pulmonary hypertension ^(61)^ (Figure 3). This context dependence highlights the need for a detailed understanding of how S1PR expression varies across different vascular beds to enable the development of more specific and effective targeted therapies.

## Conclusions

Sphingolipid signaling is a central mediator for various vascular processes. It controls VSMC proliferation and contractility and contributes to the development of atherosclerosis, pulmonary hypertension, calcification, and blood pressure homeostasis. Given the clinical availability of compounds such as fingolimod and siponimod, testing the effects of agonists or antagonists on the SPHK-S1P-S1PR axis in cardiovascular disease shows substantial clinical potential and warrants further investigation.

## Article Information

### Acknowledgments

This article is based on the study, which received the Medical Research Encouragement Prize of the Japan Medical Association in 2025. We would like to thank Editage (www.editage.jp) for English language editing.

### Author Contributions

Conceived the research topic: Junichi Saito. Contributed to the collection of related papers: Irusha Dahal and Junichi Saito. Prepared the manuscript: Irusha Dahal, Christine Sanderson, and Junichi Saito. All authors reviewed the manuscript and approved the submission.

### Conflicts of Interest

None
